# Independent Validation of Genomic Prediction in Strawberry Over Multiple Cycles

**DOI:** 10.3389/fgene.2020.596258

**Published:** 2021-01-22

**Authors:** Luis F. Osorio, Salvador A. Gezan, Sujeet Verma, Vance M. Whitaker

**Affiliations:** ^1^Gulf Coast Research and Education Center, University of Florida, Wimauma, FL, United States; ^2^School of Forest Resources and Conservation, University of Florida, Gainesville, FL, United States

**Keywords:** training population, *Fragaria*, breeding, Bayes B, genome-wide prediction, test population

## Abstract

The University of Florida strawberry (*Fragaria* × *ananassa*) breeding program has implemented genomic prediction (GP) as a tool for choosing outstanding parents for crosses over the last five seasons. This has allowed the use of some parents 1 year earlier than with traditional methods, thus reducing the duration of the breeding cycle. However, as the number of breeding cycles increases over time, greater knowledge is needed on how multiple cycles can be used in the practical implementation of GP in strawberry breeding. Advanced selections and cultivars totaling 1,558 unique individuals were tested in field trials for yield and fruit quality traits over five consecutive years and genotyped for 9,908 SNP markers. Prediction of breeding values was carried out using Bayes B models. Independent validation was carried out using separate trials/years as training (TRN) and testing (TST) populations. Single-trial predictive abilities for five polygenic traits averaged 0.35, which was reduced to 0.24 when individuals common across trials were excluded, emphasizing the importance of relatedness among training and testing populations. Training populations including up to four previous breeding cycles increased predictive abilities, likely due to increases in both training population size and relatedness. Predictive ability was also strongly influenced by heritability, but less so by changes in linkage disequilibrium and effective population size. Genotype by year interactions were minimal. A strategy for practical implementation of GP in strawberry breeding is outlined that uses multiple cycles to predict parental performance and accounts for traits not included in GP models when constructing crosses. Given the importance of relatedness to the success of GP in strawberry, future work could focus on the optimization of relatedness in the design of TRN and TST populations to increase predictive ability in the short-term without compromising long-term genetic gains.

## Introduction

The development of high throughput genotyping and new methods for analyzing genome-wide molecular data are revolutionizing crop improvement. In particular, genomic prediction (GP) is helping to increase genetic gains for genetically complex traits in animal (Hayes et al., 2009), crop (Bernardo and Yu, 2007; Crossa et al., 2010; Gezan et al., 2017), and tree breeding programs (Kumar et al., 2012; Resende et al., 2012a). Genomic prediction relies on an available set of phenotypes and DNA marker data for a training population (TRN) that is used to fit a model to predict breeding values (BV) based on DNA marker data alone for a testing population (TST). This methodology requires that the genome has been covered by a sufficiently dense panel of markers, that moderate to high linkage disequilibrium (LD) exists between marker loci and the underlying quantitative trait loci and that there is some degree of relatedness between the TRN and TST populations (Meuwissen et al., 2001).

As pointed out by Goddard (2009), LD constrains the number of markers to what is defined as “the number of chromosome segments” in a segregating population, which depends on the effective population size (*N*_*e*_). If *N*_*e*_ decreases, it is expected that the individuals within the population will share larger chromosome segments, increasing prediction accuracy (Clark et al., 2012). Moreover, as *N*_*e*_ decreases, variability on which to select will decrease, but relatedness between individuals will increase leading to greater LD in the population (Albrecht et al., 2014). Therefore, GP methods will capture both LD and relatedness among individuals in the TRN and TST populations for predictions (Habier et al., 2007; Albrecht et al., 2014). Understanding the relative impacts of LD and relatedness in a breeding program may be helpful, since LD has greater potential to persist across populations and generations (Hayes et al., 2009).

Predictive ability (PA) is defined as the correlation between the observed phenotypic value and the BV: [$r\,\left(y,\hat{g}\right)$], and prediction accuracy is the correlation between the true BV and the estimated BV, $[r\left(g,\hat{g}\right)$] (Habier et al., 2007). Different empirical equations can be used to estimate prediction accuracy for GP in one population (Daetwyler et al., 2008; VanRaden, 2008), or multiple populations, traits and environments (Wientjes et al., 2015, 2016). However, there is a concern that after several consecutive breeding cycles using GP the prediction accuracy will decline due to changes in marker allele frequency (Habier et al., 2007; Goddard, 2009), and a gradual decay of LD. Therefore, it is suggested that GP models need to be periodically re-trained to sustain long-term genetic gains (Habier et al., 2007).

Assessment of GP is not trivial. Some published studies have been based on a single population with the use of cross-validation techniques (Crossa et al., 2010; Albrecht et al., 2011; Resende et al., 2012b). Cross-validation is a statistical technique used to evaluate models where an independent dataset is not available for validation. The most common approach, in the context of GP, is the k-fold cross-validation. Here, individual observations are randomly split into five or ten subsets, and all subsets except one are used as a training population with the remaining subset serving as a validation (or testing) population in a sequential approach. Because the same original population is both part of the TRN and TST populations, predictive ability and prediction accuracy from cross-validation are often upwardly biased (Amer and Banos, 2010; Michel et al., 2016), resulting in over-optimistic models. A better alternative is to independently validate the model with another separate trial (Amer and Banos, 2010; Hofheinz et al., 2012).

Some reports on independent validation and cross-validation across environments for multiple generations using a two-stage analysis have been published (Albrecht et al., 2014; Auinger et al., 2016; Michel et al., 2016, 2017). In these studies, higher predictive abilities have been reported for cross-validation, with a TRN population sampling individuals from multiple generations and validating with an independent trial, rather than predicting from a single generation and validating with an independent trial. However, in other studies, no significant differences in predictive ability or prediction accuracy were found by using independent validation from either TRN populations constituted as cross-validation from multiple years or from single years (Sallam et al., 2015; Đorđević et al., 2019). Nevertheless, as breeding programs progress in their use of GP, independent validations will become the reference to evaluate any model.

For training populations tested across multiple environments, genotype-by-environment (G × E) interactions may be important. Several GP studies using real data under different scenarios of locations and/or environments have modeled the effects of G × E or marker × E interactions (Burgueño et al., 2012; Jarquín et al., 2014, 2017). Previous studies on genotype by location interaction (Whitaker et al., 2012) and genotype by year interaction (Gezan et al., 2017) in the strawberry (*Fragaria* × *ananassa*) production area of Central Florida have indicated either very low or the absence of G × E interaction for the main strawberry commercial traits.

The strawberry breeding program at the University of Florida (UF) conducts genetic trials at the Institute of Food and Agricultural Sciences, Gulf Coast Research and Education Center (GCREC) in Balm, FL, United States. Each year a clonally replicated field trial of advanced breeding selections is phenotyped for several polygenic traits and genotyped via single-nucleotide polymorphism (SNP) arrays. These advanced selections arose from previous marker-assisted seedling selection for simply inherited disease resistance and fruit quality traits (Roach et al., 2016; Mangandi et al., 2017; Noh et al., 2017; Salinas et al., 2019) and subsequent visual field selection of the seedlings. Yearly advanced selection trials represent the elite parent pool of the breeding program and have been used to test GP methods (Gezan et al., 2017) and to apply GP for parent selection. These accumulated trials now allow further evaluation of models in strawberry over multiple breeding cycles.

The overall objective of the present study was to inform practical approaches for the use of GP in the breeding of horticultural crops by examining multiple cycles in the UF strawberry breeding program. Our specific objectives were to: (1) examine the effects on predictive ability of combining multiple cycles (or years) into TRN populations in the forward and backward directions; and (2) examine the effects of relatedness among the TRN and TST populations, LD and *Ne* on changes in predictive ability over time.

## Materials and Methods

### Population and Field Testing

The elite population of the UF strawberry breeding program is treated as a single breeding pool from which the top-ranked parents of the previous year are used in a partial circular mating design to generate a large population of seedlings to be evaluated. This mating design is a modification of a partial diallel design with a reduced number of four to five crosses per parent, that fall along an off-diagonal matrix of parental crosses (White et al., 2007). The best seedling selections are established the following year in an advanced-selection trial, the structure of which consists of a mixture of full-sib families, half-sib families, advanced selections, and cultivars. A representation of the structure of the population across cycles is presented in Table 1.

**TABLE 1:** Incidence matrix for common genotypes tested among trials (above diagonal), full-sib families (diagonal, in bold) and common parents of full-sib families among trials (below diagonal).

**Trials**	**T2**	**T4**	**T6**	**T8**	**T10**	***N***
T2	**33**	37	29	28	30	217
T4	8	**30**	57	40	43	240
T6	2	7	**45**	88	69	237
T8	3	1	14	**43**	107	273
T10	2	3	10	13	**28**	266

*N is the total number of tested phenotypes excluding common genotypes across trials. The T2–T10 nomenclature for the five trials conducted in five successive years is according to Gezan et al. (2017).*

Replicated seedling and advanced-selection trials were previously established at two sites, the Gulf Coast Research and Education Center (GCREC) in Balm, FL (lat. 27° 45′ 37.98″ N, long. 82° 13′ 32.49″ W) and at the Florida Strawberry Growers Association in Dover, FL (lat. 28° 0′ 55.55″ N, long. 82° 14′ 5.24″ W), during the 2013–2014 and 2014–2015 seasons. Very low genotype by location interactions were observed for yield and quality traits (Whitaker et al., 2012). Consequently, these trials were subsequently carried out only at the GCREC.

The populations included in the present study were established at the GCREC site during five consecutive seasons from 2013–2014 to 2017–2018. The strawberry breeding program uses an overlapping generation breeding strategy in which all the main breeding activities, crossing, testing, and selection, take place each year (Borralho and Dutkowski, 1998), therefore each trial was considered a cycle in this sense and was given an even-numbered code starting with season 2013–2014 as T2 and ending with 2017–2018 as T10 according to the naming convention of Gezan et al. (2017). Several common genotypes were tested across years including cultivars and advanced selections chosen for further testing in the breeding process (Table 1). Therefore, these are essentially independent trials established under different yearly environmental conditions. Seedlings were clonally propagated by runners in a summer nursery near Monte Vista, Colorado (T2 and T4 trials) and at Crown Nursery in Malin, Oregon (T6, T8, and T10) and established in the fruiting field at GCREC in the first 2 weeks of October in each year. Site preparation, trial establishment and trial maintenance was carried out according to standard commercial practices for west-central Florida (Torres-Quezada et al., 2018). Pest control, fertilization and weed control varied among seasons according to environmental conditions. Bare-root clonal plants were arranged in a randomized complete block design with either five or six replications per trial and raised beds within replication. Each bed was subdivided into five to nine plots, each with a common control genotype to account for environmental variation along the bed. Genotypes were represented by a single runner plant in each plot (Supplementary Table S1).

### Phenotyping and Genotyping

Five yield and fruit quality traits were assessed weekly from mid-November to mid-March in all five trials. At each harvest date, all ripe fruit per plant was removed. All marketable fruit (grams) by plant were considered as early marketable yield (EMY) if harvested before the first day of February. Total marketable yield (TMY) was calculated as the marketable fruit by plant collected until the first week of March. Average fruit weigh in grams, AWT, was estimated as the TMY divided by the number of marketable fruit. Total culls (TC), or unmarketable fruit, were counted and expressed as a proportion of the total number of fruits per plant (%). Soluble solids content (SSC) was measured five times during the season in each trial and was calculated as the mean of all measurements. One ripe fruit from each plant was squeezed by hand onto a handheld digital refractometer.

There were a total of 1,715 entries planted in these five trials that were phenotyped and genotyped using the Affymetrix Axiom^®^ IStraw90 (Bassil et al., 2015) and IStraw35 (Verma et al., 2017) SNP arrays. Quality control was performed on a total of 14,332 segregating SNP markers in which SNPs with MAF < 0.05, and missing marker data >0.05 were eliminated, yielding a total of 9,908 markers for the analyses. Missing values for each of the markers were imputed based on average allele frequency. The 1,715 phenotypes represented 1,558 unique individuals including advanced selections and varieties that were repeated across trials.

### Genomic Prediction Model Analyses

The GP approach implemented was based on best linear unbiased estimates (BLUE) following one-stage analysis of tested phenotypes adjusted for the experimental factors in each trial. In most years, row and column location of each plant in the trial was recorded and the general linear mixed model was modified by adding spatial factors (row, col) and correlated residuals (autoregressive of order 1 for row and column), or independent residual units. Hence, multiple linear mixed models were tested for each trait and evaluated based on the Akaike and Bayesian information criteria (AIC and BIC, respectively) as well as their numbers of parameters (Isik et al., 2017).

Genomic Best Linear Unbiased Prediction, GBLUP (VanRaden, 2008) allowed the testing of complex models and was used only to assess genotype by year interactions (G × Y) between pairs of years and calculate heritabilities. The multi-year model assumed the genotypes among years were correlated such that genetic correlations could be estimated among years, using a factor analytic variance-covariance structure with two unknown factors (as fully described by Smith et al., 2001). Factor analytic models have been used to a large degree in plant breeding programs to model G × E interaction with heterogeneous variances between environments, and have shown to work well for crop species in multi-environment tests (for example, Burgueño et al., 2007, 2012; Crossa et al., 2006; Oakey et al., 2016; Dias et al., 2018). We used a multivariate model with a factor analytic variance-covariance structure with two (*K*) unknown factor loadings. When the factor analytic model is applied to the matrix of genotypic effects in each year (*u*_*g*_), the model can be written as: *u*_*g*_ = (Γ⊗*I*_*m*_)*f*+δ,where Γ is the matrix of *K* vector loadings, *f* is a vector of genotypic scores; *I*_*m *_is the vector of genotypes in each year and δ is the vector of genetic regression residuals. The variance of the genotype effects by year takes the form: *var*(*u*_*g*_) = (Γ Ѓ + ψ)⊗*I*_*m*_where ψ is a diagonal matrix with ψ_*i*_ as the specific variance for the *i*^*th*^ year, and the matrix across years is *G* = (Γ Ѓ + ψ).

In this analysis, a genomic relationship matrix *G* was generated using all 9,908 markers and following the methodology described by Yang et al. (2010). The *G* matrix and its inverse were performed with the software GenoMatrix (Nazarian and Gezan, 2016), and model fitting was carried out with ASReml-R version 4.0 (Butler et al., 2017) R version 3.5.1 (R Core Team, 2018).

Genomic prediction models, for this study, were obtained by Bayes B and GBLUP, however, Bayes B has been shown to capture both marker-quantitative trait loci association effects and genetic relationship effects better than BLUP methods (Zhong et al., 2009). Even though, GBLUP has indicated to have a good performance for real data application (de los Campos et al., 2013), in a previous strawberry prediction study (Gezan et al., 2017). Bayes B performed slightly better for low-heritability traits and was therefore the main focus in our estimation of predictive ability for each TST population. In Bayes B, the analysis of each trait within each year was performed according to the following mixed model:*y* = 1μ+*Z*β+*e*, where *y* is the response vector of BLUES, μ is the intercept, β is a vector of random marker effects (coded 0, 1, 2) associated with the incidence matrix *Z* and *e* is the vector of residual effects. Bayes B is a variable selection and shrinkage method, which assumes that some SNP effects are non-zero with probability 1-π while others have zero effects with probability π, following a mixture of two different prior densities with a point of mass at zero and a slab with a scaled-*t* density (de los Campos et al., 2013). In this study, we defined the priors according to the default hyper-parameters recommended by Pérez and de los Campos (2014).

We estimated predictive abilities by fitting the model for each trait with data from each individual trial as a training set (e.g., T2) and predicting to other trials (or years), as testing sets (e.g., T4, T6). Therefore, when we used T2 as TRN population we made a prediction for all T4 to T10 trials, by employing a single matrix of marker effects. The genotypes in these trials are genetically related to various degrees, but they are statistically independent in the process of fitting and evaluating the genetic model. After the single predictions were performed, we increasingly averaged successive predictions from previous years to the latest cycle (T10) and evaluated their effect on predictive ability in both forward (T2, T24,…) and backward (T8642, T864,…) directions. Each of these combinations was evaluated including or excluding common genotypes trialed across years. The Bayes B model was fitted in R (R Core Team, 2018) using the R package BGLR (Pérez and de los Campos, 2014) implementing a Markov Chain Monte Carlo method with 50,000 iterations where the first 10,000 were used as a burn-in. Each trait in each year was run five times and the predictive ability (PA) was estimated as the average of all runs, and trace plots of the residual variance were checked. The heritability of adjusted clonal mean phenotypes was estimated using GBLUP, with and without common genotypes, as $h^{2}=\frac{\sigma_{a}^{2}}{\sigma_{a}^{2}+\sigma_{e}^{2}},$ where $\sigma_{a}^{2}$ is the additive variance and $\sigma_{e}^{2}$ is the estimated residual variance. Even though there was a moderate number of full-sib families in each trial (Table 1), we did not estimate within-family predictive ability for each cycle because of the unbalanced and small number of seedlings per family, mostly varying between 3 and 10. Within-family predictive ability is estimated in a different study (in preparation) established for three consecutive years with few biparental crosses and a large number of seedlings per family (60–75).

### Linkage Disequilibrium and Effective Population Size

The previously mentioned set of 9,908 SNP markers was used to estimate effective population size, *Ne.* This set of markers was selected out of 14,332 markers in season 2015–2016 using the GenoMatrix software (Nazarian and Gezan, 2016) and was used for all other GP analyses. A closely related set of 9,622 genetically mapped SNP markers from Axiom IStraw35 SNP array (Verma et al., 2017) were used to estimate linkage disequilibrium (LD) for the five trials – T2, T4, T6, T8, and T10. These markers were distributed among 28 linkage groups (LGs) with a minimum number of 15 markers and maximum number of 720 markers per LG (Supplementary Table S2). The multi-year dataset comprising all cycles was divided into five different subsets based on crossing year. The purpose of dividing datasets this way was to estimate the distribution of LD structure and *Ne* of each trial without the genetic background influence of parents and common genotypes among trials. All individuals from T2 were included: parents, selections, and ancestors connected to the rest of the trials. Datasets for subsequent cycles T4, T6, T8, and T10 for the purposes of LD and *Ne* estimation included no founders or check cultivars, as the inclusion of common individuals across trials might influence haploblock structure estimation.

The R packages synbreed (Wimmer et al., 2012) and LDcorSV (Desrousseaux et al., 2017) were used to estimate LD based on population relatedness (*r*^2^) and without relatedness (*r^2^_*v*_*), respectively (Mangin et al., 2012). The LD decay in genetic distance (Mb) was fitted with a non-linear regression model within the synbreed package. *Ne* was estimated using an LD-based approach and allele frequency threshold of 0.05 (Waples, 2006) via NeEstimator v2.1 software (Do et al., 2014). NeEstimator V2.1 (2017) is a tool for estimating contemporary effective population size (Ne) using multi-locus diploid genotypes from population samples. Unlike V1, NeEstimator V2.1 does not include third-party programs; all methods are implemented by NeEstimator V2.1 code and also implements a bias-corrected version of the method based on linkage disequilibrium (LD).

## Results

### Training GP Models With Multiple Cycles

**FIGURE 1:**
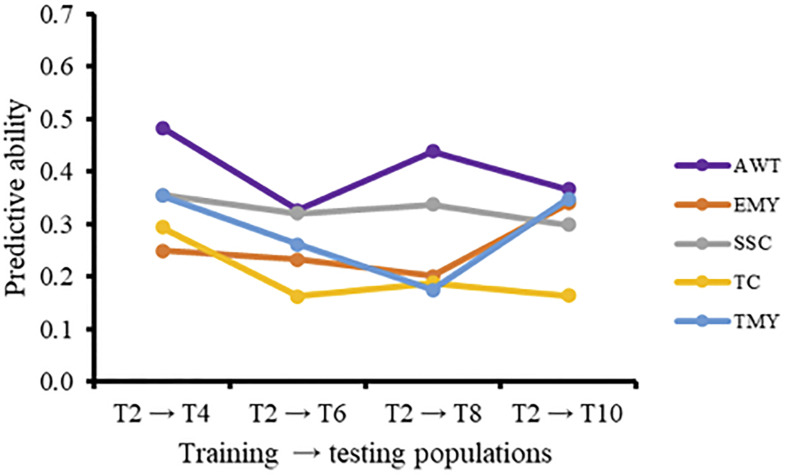
Predictive ability (PA), without common genotypes and varieties, using T2 as an independent training population to predict later cycles for five traits. AWT, average fruit weight (g); EMY, early marketable yield (g per plant); SSC, soluble solids content (°Brix); TC, proportion of total culls (%); TMY, total marketable yield (g per plant).

**FIGURE 2:**
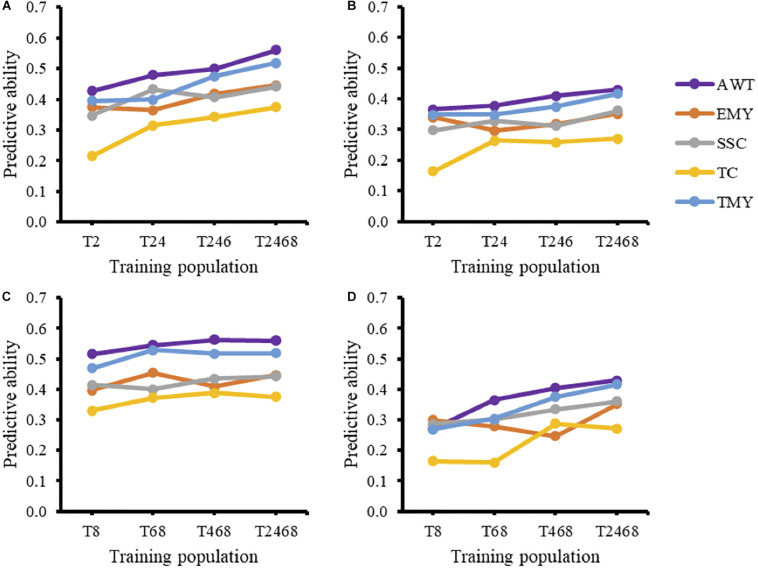
Forward **(A,B)** and backward predictions **(C,D)** of trial T10 (testing population) and the effect of model averaging the training population on predictive ability under two scenarios: including common genotypes between the training and testing populations **(A,C)** and excluding them **(B,D)**. AWT, average fruit weight (g); EMY, early marketable yield (g per plant); SSC, soluble solids content (°Brix); TC, proportion of total culls (%); TMY, total marketable yield (g per plant).

The effect of using a GP model over multiple breeding cycles without retraining can be seen when using T2 as a training population for all successive cycles (Figure 1). For all traits except EMY there was a negative trend in predictive ability over time. The increase in predictive ability of EMY and TMY from cycle 2 to cycle 3 seems to be associated with an increase in heritability, from the TRN to the TST population, that was not present in other traits. The inclusion of additional cycles to the training population in the forward direction for prediction of trial T10 resulted in increased predictive abilities (Figures 2A,B). Predictive abilities for AWT and TMY tended to increase continuously, whether common genotypes across trials were included or not, while the trends for the other traits were more variable, but still showing an overall positive trend.

Predictive abilities were noticeably higher when common genotypes were included across cycles (Figure 2), and in this scenario backward predictions had on average higher predictive abilities for all traits than forward predictions. When common genotypes were included in the analyses, adding additional cycles to the training population in the backward direction gave little improvement. For example, there seemed to be no improvement in predictive ability when trial T2 was added to a training population consisting of trials T8, T6, and T4. However, when common genotypes were excluded, the addition of cycles to the training population in the backward direction noticeably improved predictive abilities for most traits.

### Genetic Relationships

Single-cycle predictive abilities based on Bayes B are depicted in Table 2. The scenario in which all common genotypes between TRN and TST populations were included had a higher average predictive ability (0.35) than for the scenario excluding common genotypes (0.24), as expected. The trait AWT, when common individuals were included, had the highest average PA (0.43) of all traits across cycles, with a range from 0.38 to 0.53, followed by SSC (0.38), TMY (0.35), EMY (0.30), and TC (0.28). A similar pattern was noted when excluding common individuals, where AWT had the highest average PA (0.33) varying from 0.15 to 0.48, followed by SSC (0.26), TMY (0.24), EMY (0.18), and TC (0.18). The predictive abilities estimated by Bayes B and GBLUP were very similar (Table 2 and Supplementary Table S4).

**TABLE 2:** Forward predictive ability (PA) for five traits estimated using Bayes B, for pairs of trials using: (A) all individuals including varieties and advanced selections in common among each pair of trials, and (B) excluding common individuals.

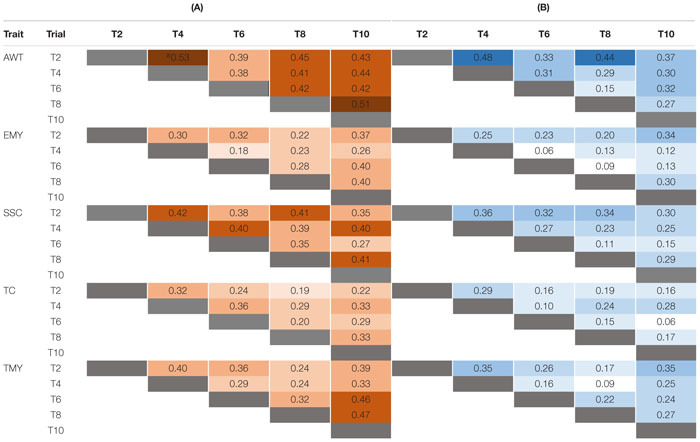						

*AWT, average fruit weight (g); EMY, early marketable yield (g per plant); SSC, soluble solids content (%); TC, proportion of total culls (%); TMY, total marketable yield (g per plant). ^*a*^Predictive ability ranges from low (light color) to high (dark color).*

### Heritabilities and G × E Interaction

**FIGURE 3:**
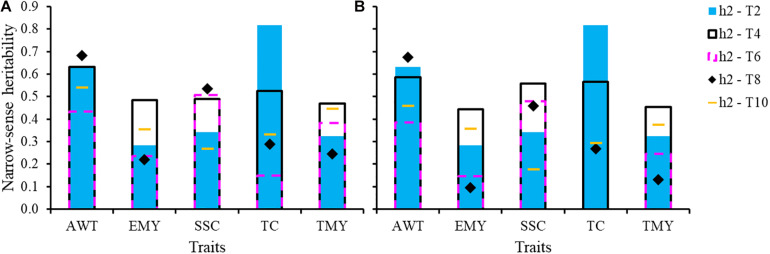
Genomic narrow-sense heritabilities for five traits for each trial with: **(A)** all genotypes including varieties and advanced selections in common among pairs of trials, and **(B)** excluding common genotypes. AWT, average fruit weight (g); EMY, early marketable yield (g per plant); SSC, soluble solids content (°Brix); TC, proportion of total culls (%); TMY, total marketable yield (g per plant).

Genomic heritability estimates are presented in Figure 3. Heritability estimates excluding common genotypes among trials between TRN and TST were lower than those estimates including common individuals across trials in 80% of the cases. However, the range of heritabilities in both scenarios was wide and similar, whether excluding or including common individuals, mostly varying from 0.15 to 0.65, except for the wider range for TC (0.0–0.81). Overall, average additive genetic correlations across trials were very high, indicating very little if any G × Y interaction (Table 3). Though a few values in some cycles showed moderate correlations, such as for EMY (0.70) and TC (0.72), all remaining values were higher than 0.79 (Supplementary Table S3).

**TABLE 3:** Average additive genetic correlations for five traits across trials, including common individuals among trials, using GBLUP and a factor analytic of order 2 (FA2) variance-covariance matrix, together with the proportion of the total genetic variance explained (VE%) by FA2.

***r*_*a*_**	**AWT**	**EMY**	**SSC**	**TC**	**TMY**
Mean	0.96	0.95	0.9	0.9	0.95
Range	0.94–1.00	0.69–1.00	0.87–1.00	0.72–1.00	0.86–1.00
VE%	97.9	100.0	97.3	98.9	100.0

*AWT, average fruit weight (g); EMY, early marketable yield (g per plant); SSC, soluble solids content (%); TC, proportion of total culls (%); TMY, total marketable yield (g per plant).*

### Linkage Disequilibrium and Effective Population Size

**FIGURE 4:**
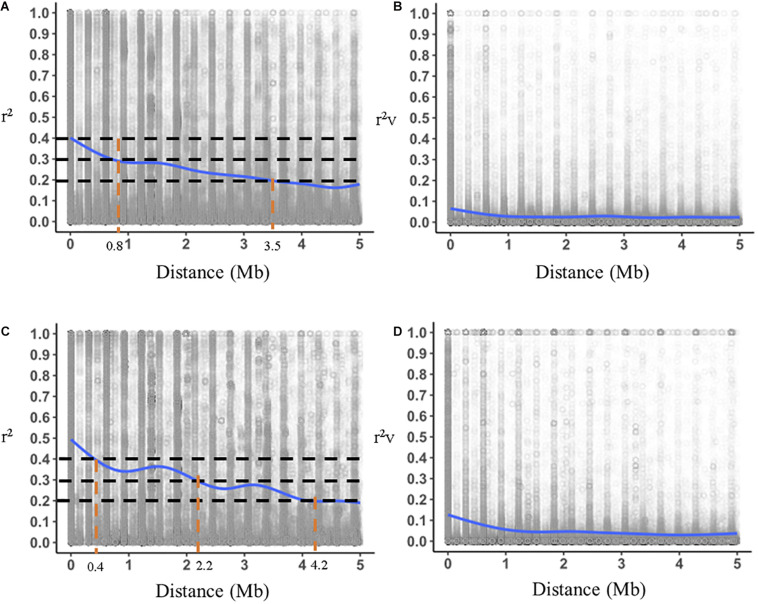
Comparison of genome-wide decay of r^2^ (linkage disequilibrium with relatedness bias) **(A,C)** and r^2^v (linkage disequilibrium without relatedness bias) **(B,D)** for the T2 (Cycle 0) and T10 (Cycle 4) trials. Horizontal dotted lines represent thresholds of r^2^ for comparison and vertical dotted lines represent genomic distances where LD intersects with thresholds.

A set of 9,622 markers were mapped to 40 linkage groups, the number of markers per LG varying from 15 to 720. We plotted *r*^2^ and *r^2^v* (*r*^2^ with no relatedness bias) for T2 and T10 against genomic distances in Mb for T2 and T10 (Figure 4). We also compared the decay of LD between T2 and T10. Maximum *r*^2^ was 0.4 in T2 and 0.47 in T10. In T2, *r*^2^ decreased to 0.2 at 3.5 Mb (Figure 4A), compared to an *r*^2^ of 0.2 at 4.2 Mb for T10 (Figure 4C). Similar trends were observed for *r^2^v*, with a slower decay of LD in T10 compared to T2 (Figures 4B,D). Much higher values overall for *r*^2^ compared to *r^2^v* indicates that a substantial portion of apparent LD was due to relatedness (Supplementary Table S2). The effective population sizes, *Ne*, for each of the cycles were 25, 17, 23, 23, and 20 for T2, T4, T6, T8, and T10, respectively, possibly indicating a slight decrease over time.

## Discussion

Independent validation with TRN populations from five breeding cycles was utilized to evaluate GP methods and inform practical approaches for its implementation in the strawberry breeding program at UF. The impact of averaging multiple single predictions, genetic relationships among the cycles, heritabilities, G × Y interactions, LD and *Ne* were explored separately. The estimation of trait additive correlations across years, G × Y, using multivariate analyses is complex due to the heterogenous variances-covariances among environments and the environmental effects to be fitted. When the number of traits is high using a parsimonious FA matrix in modeling the G × Y interaction has advantages in convergence compared to models using an unstructured variance-covariance matrix. Previous results showed that increasing the number of components of FA models would give better estimates of variance-covariance estimates; however, these models may or may not increase predictive ability, and it is questionable whether it would improve the model fit (Burgueño et al., 2011). Though our estimates of additive correlations across years (Table 3) might be upwardly biased, they reflect the low G × Y interactions present for the traits evaluated.

Our focus on the estimation of predictive abilities was due to the primary emphasis in this study on practical outcomes and applications; however, it is possible to use deterministic formulae to calculate prediction accuracies between different cycles, which we would expect to provide very similar trends (Wientjes et al., 2015). Prediction accuracy and the reliability of predictions has been shown to decline across generations due to a decrease in genetic relationships between the TRN and TST populations (Habier et al., 2007; Pszczola et al., 2012) as well as the break-up of LD and consequent reduction of genetic variance explained by the markers (Goddard, 2009). Therefore, retraining models for GP is recommended every generation (Wolc et al., 2011; Pszczola and Calus, 2016). Currently, in the UF strawberry breeding program the decay of predictive ability over successive cycles without including common individuals (Figure 1) is offset by updating the GP model every year with phenotypic and marker data from the latest field trial. Besides, significant decreases in selection accuracy over generations are not expected if marker density is sufficiently high (Solberg et al., 2008). The number of markers used in this set of trials (∼10,000) might be considered small when compared with some other breeding programs, particularly for animals. However, the most complete strawberry genetic map developed for UF germplasm (unpublished) has a total length of 1729.5 cM, meaning that on average more than five markers per cM were utilized in this study, which should be more than enough to account for genome-wide allelic diversity in an elite strawberry breeding population.

The results obtained by comparing predictive abilities estimated by Bayes B, as well as a previous report using different methods of predictions (Gezan et al., 2017), indicate that, for the commercial traits reported, Bayes B may produce slightly greater predictive abilities than GBLUP. Therefore, we are using Bayes B operationally in the breeding program and have focused on the use of Bayes B for this report. Overall, predictive abilities using single cycles (or trials) as training populations (Table 2) were in the general range of estimates reported from other crops and environments (Sallam et al., 2015; Đorđević et al., 2019). Using multiple cycles by averaging predictions across cycles noticeably increased predictive ability, whether individuals common to multiple trials were included in the analyses or not. Thus, the size of the training population, which is known to be important for the success of GP, was increased, not in the traditional sense (Asoro et al., 2011; Zhang et al., 2017), but with the addition of independent training populations from each cycle. Improvements in the estimation of PAs by adding multiple cycles of training populations could also come from averaging G × Y interaction effects, though we have shown these to be quite low (Table 3).

The presence of population structure across the breeding cycles has important effects on GP (Asoro et al., 2011). Genetic relationships in the strawberry breeding populations studied arise from two primary sources: the first is the continued testing across years of promising advanced selections and check cultivars during the process of variety development, and the second is the use of common parents across years which increases relatedness at the half-sib family level (Table 1). The impacts of genetic relationships and cosegregation can be seen by comparing the structure of the TRN populations in Table 1 with the predictive abilities in Table 2 when including common individuals and when excluding them. As shown in Table 1, the average number of common genotypes among T2 or T4 with the other trials is 31 and 44 genotypes, respectively. Among the T6, T8, and T10 trials the average number of common individuals with others is 61, 66, and 62, respectively, partly reflecting the larger number of genotypes included in these later trials. This helps to explain the increasing average differences in predictive ability across traits over time between scenarios where common individuals are included versus excluded: T2 (0.05), T4 (0.12), T6 (0.18), and T8 (0.17). Common parents as a source of relatedness is highlighted by the fact that the average number of parents shared among individuals for either T2 or T4 with the other trials is four and five, respectively, but for T6, T8, and T10 trials the average number of shared parents is eight, eight and seven, respectively. In other words, the increase in genetic relationships across cycles over time is clearly one of the factors favoring predictive ability in this breeding program.

The strength of family relationships within and across populations has been shown to influence the reliability and the accuracy of genomic predictions in several studies. In Pszczola et al. (2012) the effect of four TRN populations with increasing numbers of half-sib families (5, 20, 40) for a fixed number of offspring and a random population with the same number of individuals was simulated. Based on their results and other studies (Calus, 2010), the authors concluded that highly related TRN populations that have a small number of families with large number of offspring per family yield lower accuracy of prediction compared to TRN populations with more half-sib families or random populations. In the UF strawberry breeding program the composition of the TRN population is largely determined by the field performance of seedlings selected in the previous year. Different numbers of seedlings are selected from each full-sib family based on performance, while also aiming to have, if possible, all families represented to maintain genetic diversity. This resulted in small and unbalanced numbers of individuals representing each full-sib family, which is why within-family predictions were not performed in this study. Ultimately, optimizing the design of the TRN population at the family level is achievable, but constraining the number of selections in the best families may negatively affect genetic gains, at least in the short-term. The increase from two common parents between T2 and T10 to 13 common parents between T8 and T10 might have had a positive effect on predictive ability. Yet this is not obvious, since in the scenario of excluding common individuals the predictive ability for all traits from T8 to T10 (Figure 2D) was lower than the predictive ability from T2 to T10 (Figure 2B), indicating the low impact of the number of half-sibs in this scenario. When including common individuals, the situation is reversed, with T8 having greater ability than T2 to predict T10. It is also important to note that backward predictions when common individuals are included quickly reach a plateau, with the addition of T6 to T8 giving a very small increase in PA and the addition of T4 and T2 giving no improvement (Figure 2C). Together these results highlight the importance of relatedness to predictive ability, particularly in the case of common individuals.

Marker-based genomic heritability estimates from this study are higher than the previously reported pedigree-based estimates for T2 and T4 (Gezan et al., 2017). This is not surprising, as marker-based relationships are more precise. Many studies have shown positive correlations between predictive ability and narrow sense heritability, consistent with the present study (Calus et al., 2008; Daetwyler et al., 2008). The presence of G × Y interactions may cause rank changes across years, when pairwise genetic correlations among years are below *r*_*a*_ = 0.8 (White et al., 2007; Goddard and Hayes, 2007). In this study, almost all additive correlations were above 0.8, suggesting low G × Y interactions that will have little effect on PA. Most of the strawberry production in Florida is concentrated within a 30-mile radius of Plant City, and genotype by location interaction is minimal within this region. On the other hand, G × Y is more unpredictable and should be monitored closely over time. Modeling G × Y could allow trials to be pooled into a single training population, as opposed to averaging predictions across cycles, possibly improving PA.

Estimates of intra-linkage group regular pairwise LD (*r*^2^) and LD corrected for relatedness (*r^2^_*v*_*) for T2 were slightly lower than our previous estimates of *r*^2^ = 0.26 and *r^2^_*v*_* = 0.04 (Gezan et al., 2017). One possible reason is that the original study utilized 17,479 markers from the IStraw90 SNP array, while the present analysis was based on 9,622 markers from the IStraw35 array (Verma et al., 2017) which also provides the same quality of data but at a reduced cost. Simulation studies have shown that overestimation of LD (*r*^2^) comes first from multiples copies of the same genotype and second from the progeny of full-sib families (Mangin et al., 2015). In our analysis, we estimated *r*^2^ based on a single copy of each phenotype (common individuals removed), but there were multiple full-sib families with different numbers of offspring in each cycle; therefore, the bias of the *r*^2^ estimate should only be due to this second factor. The presence of LD corrected for relatedness is the driving force for the long-term success of GP in the breeding population, as *r^2^_*v*_* represents the prediction accuracy that will tend to persist over multiple cycles without the need for retraining (Mangin et al., 2012; Habier et al., 2013). The dramatic decrease in LD when removing relatedness bias once again emphasizes the importance of relatedness in this population as it relates to the success of GP models.

The impact of *Ne* on prediction accuracy has been reported in animals, forest trees and tree fruit species (Kumar et al., 2012; Daetwyler et al., 2013; Bartholome et al., 2016). In long generation tree species, the use of elite populations with *Ne* ranging from 10 to 50 is a common practice to increase genetic gains. In this study, effective population size appears to have decreased slightly from T2 (*Ne* = 25) to T10 (*Ne* = 20). In the present study this apparent slight reduction in *Ne* and the corresponding increase in the extent of LD from T2 to T10 are likely contributing to increased predictive ability with the addition of later cycles. In the long-term it is important to recognize that intensive recurrent selection increases inbreeding. Therefore, to maintain long-term breeding progress, it will be important to continue to introgress diversity into the elite breeding population.

**FIGURE 5:**
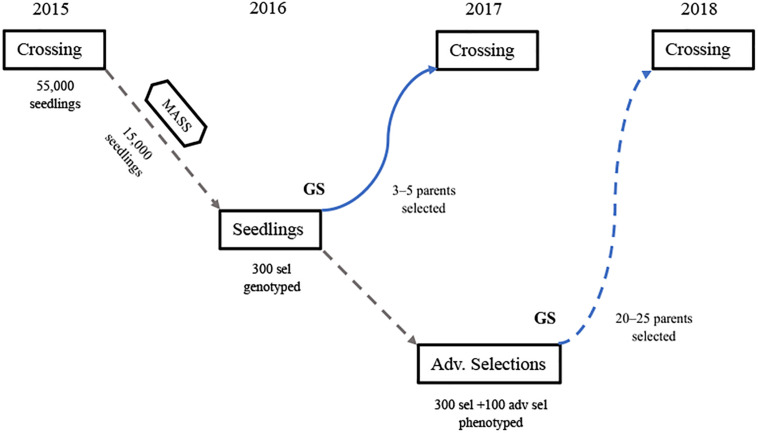
A single UF strawberry breeding cycle (overlapping cycles not shown) combining marker-assisted seedling selection (MASS) for disease resistance and other simply controlled traits, combined with the early implementation of GP for using untested genotypes in crosses 1 year early (blue line), prior their phenotyping in a replicated field trial of elite clones (GP trial). Once all clones are phenotyped, GP models are updated with that information for the estimation of BVs to guide future crosses (dashed line). For ease of visualization, this figure shows only the cycle beginning in 2015. However, due to yearly overlapping cycles, all breeding program activities including crossing, MASS, seedling trials, GP trials, etc., are carried out every year.

The last 5 years of implementation of GP in the UF strawberry breeding program has allowed the use of some parents earlier in the breeding cycle and has increased the accuracy of estimation of breeding values. This study makes clear that the use of average predictions from multiple cycles in training GP models is very beneficial, at least up to four cycles when common individuals are included across trials. Based on these results, the following steps are currently used for the application of GP in the UF strawberry breeding program (Figure 5):

(1) In the summer prior to each winter fruiting/crossing season, which in Florida extends roughly from mid-November through March, phenotypic and marker data from up to four previous cycles, including common individuals across trials, are used to train Bayes B models predicting the BVs of the most recent advanced selections. These selections were seedlings in the previous cycle and are genotyped over the summer but are not yet phenotyped for the five measured commercial traits AWT, EMY, SSC, TC, and TMY.
(2) Breeding values for these five traits are combined in a selection index using economic weights for each trait to rank the advanced selections for their overall potential as parents.
(3) In November and December, early-season field observations are made for these advanced selections for all visually evaluated traits, including: fruit shape, color, and flavor, disease resistance, plant architecture, etc.
(4) Three to five advanced selections (out of approximately 25–40 total parents) that are noted for early-season field traits and ranked highly in the BV selection index are selected for use as parents in controlled crosses as males. These males are crossed to one or more elite females that have been field evaluated for multiple seasons and have complementary traits to the males chosen by GP. In this way, approximately 10% of crosses have a male parent chosen via GP methods that is being used in crossing at least 1 year earlier in the breeding cycle than normal.

As this study suggests, increasing the size of the training population will increase prediction accuracy, but at some point, increasing size will not further improve GP models. This appears to have occurred for the UF strawberry breeding program at the fourth cycle. Given the demonstrated importance of relatedness in this study, future work on the optimal design of the relatedness within and among TRN and TST populations (choosing which genotypes to establish in each trial) could possibly increase predictive ability in the short term without compromising the potential of future genetic gains. It will also be important to monitor the performance of crosses chosen via GP versus those designed in the traditional manner to empirically test whether the implementation of GP in the breeding program is achieving the desired results.

## Data Availability Statement

The datasets presented in this study can be found in Data Dryad via the following link: https://datadryad.org/stash/dataset/doi:10.5061/dryad.b5mkkwhc7.

## Author Contributions

VW, SG, and LO conceived and designed the study. SV prepared the SNP data for the GP analyses and carried out the analyses of LD and Ne. SG and LO performed the GP analyses. LO wrote the initial draft and VW, SG, and SV corrected it and improved it. All authors read and approved the manuscript.

## Conflict of Interest

The authors declare that the research was conducted in the absence of any commercial or financial relationships that could be construed as a potential conflict of interest.
