# Supplementary material for: Independent Validation of Genomic Prediction in Strawberry Over Multiple Cycles
Source: Front Genet. 2021 Jan 22;11:596258. doi: 10.3389/fgene.2020.596258 (PMC7862747; doi:10.3389/fgene.2020.596258)
Supplement: Supplementary file 1 [file Data_Sheet_1.PDF]

Supplementary Materials

Supplementary materials include supplementary Tables S1, S2, S3 and S4.

Supplementary Table S1**.** Field trial layout across cycles (years) showing the number of tested clones (N), including cultivars and advanced selections, phenotyped and genotyped.

Season	[a] Cycle	[b] Trial	N	Reps	Beds	Plots
2013-14	0	T2	217	5	1	7
2014-15	1	T4	277	5	1	9
2015-16	2	T6	299	6	1	8
2016-17	3	T8	371	5	2	5
2017-18	4	T10	394	5	2	5

[a] Cycle 0 is the most diverse trial and reference point for comparison of following cycles
[b] Naming convention for trials follows Gezan et al. (2017).

Supplementary Table S2. Linkage group-wise linkage disequilibrium (LD) (r^2) for T2, T4, T6, T8, and T10 trials and, for comparison, LD adjusted for relatedness (r^2v) for T10. Number of SNPs per linkage group, length of each linkage group (bp), and average distance between SNPs per linkage group are also presented. The last row depicts average values except for totals in the case of length of linkage group and number of SNPs per linkage group.

LG	Subgenome	No. SNPs	Length (bp)	AvDist (bp)	Mean r^2 T2	Mean r^2 T4	Mean r^2 T6	Mean r^2 T8	Mean r^2 T10	Mean r^2v T2	Mean r^2v T10
1	1A	67	9,881,146	149,714.30	0.26	0.24	0.22	0.30	0.26	0.04	0.06
1	1AII	369	11,707,472	31,813.78	0.25	0.35	0.33	0.29	0.37	0.04	0.06
2	1B	427	67,600,548	158,686.70	0.14	0.11	0.10	0.14	0.11	0.03	0.08
3	1C	40	57	1.46	0.76	0.87	0.90	0.87	0.94	0.1	0.33
3	1CII	86	20,023,073	235,565.60	0.22	0.23	0.22	0.17	0.22	0.02	0.04
4	1D	62	18,934,074	310,394.70	0.18	0.23	0.24	0.19	0.24	0.03	0.06
4	1DII	44	17,176,037	399,442.70	0.16	0.15	0.20	0.19	0.22	0.02	0.07
5	2A	87	10,409,090	121,035.90	0.3	0.35	0.40	0.33	0.35	0.03	0.06
5	2AII	109	12,615,122	116,806.70	0.2	0.24	0.22	0.24	0.26	0.03	0.05
6	2B	140	12,874,164	92,619.88	0.45	0.48	0.46	0.43	0.45	0.04	0.07
6	2BII	54	13,600,058	256,604.90	0.3	0.32	0.31	0.32	0.37	0.03	0.05
7	2C	100	15,356,138	155,112.50	0.23	0.30	0.26	0.26	0.26	0.03	0.05
7	2CII	273	28,480,348	104,707.20	0.11	0.16	0.20	0.15	0.16	0.03	0.07
8	2D	59	3,049,085	52,570.43	0.4	0.47	0.60	0.57	0.65	0.04	0.10
9	3A	240	25,074,337	104,913.50	0.2	0.23	0.24	0.21	0.24	0.02	0.03
9	3AII	103	14,189,135	139,109.20	0.17	0.17	0.18	0.15	0.15	0.03	0.04
10	3B	269	61,848,369	230,777.50	0.12	0.15	0.17	0.13	0.14	0.01	0.03
11	3C	150	36,828,167	247,168.90	0.16	0.23	0.20	0.16	0.20	0.03	0.04
11	3CII	362	57,007,392	157,915.20	0.17	0.20	0.21	0.20	0.18	0.02	0.03
12	3D	630	136,420,767	216,885.20	0.06	0.07	0.07	0.06	0.06	0.01	0.01
13	4A	45	17,072,042	388,001.00	0.35	0.52	0.56	0.39	0.48	0.04	0.08
13	4AII	15	6,213,011	443,786.50	0.12	0.11	0.14	0.10	0.17	0.02	0.08
14	4B	50	17,421,053	355,531.70	0.22	0.42	0.33	0.28	0.39	0.04	0.06
14	4BII	134	30,195,148	227,031.20	0.08	0.11	0.09	0.09	0.10	0.02	0.03
15	4C	173	74,139,184	431,041.80	0.08	0.08	0.09	0.09	0.09	0.01	0.02
16	4D	185	48,653,208	264,419.60	0.11	0.12	0.13	0.11	0.12	0.01	0.02
17	5A	312	50,289,391	161,702.20	0.09	0.12	0.12	0.11	0.12	0.02	0.02
18	5B	63	13,512,073	217,936.70	0.13	0.16	0.19	0.13	0.18	0.03	0.05
18	5BII	178	17,819,252	100,673.70	0.2	0.26	0.26	0.19	0.22	0.03	0.05
19	5C	516	133,659,683	259,533.40	0.06	0.09	0.08	0.07	0.08	0.01	0.02
20	5D	602	100,517,774	167,250.90	0.06	0.08	0.07	0.06	0.08	0.01	0.02
21	6A	720	91,844,844	127,739.70	0.08	0.10	0.10	0.09	0.11	0.02	0.02
22	6B	558	121,086,671	217,390.80	0.08	0.10	0.09	0.08	0.08	0.02	0.02
23	6C	281	21,336,382	76,201.36	0.14	0.17	0.15	0.16	0.16	0.02	0.02
23	6CII	190	18,805,240	99,498.62	0.33	0.34	0.34	0.33	0.30	0.04	0.05
24	6D	296	74,881,388	253,835.20	0.08	0.09	0.11	0.09	0.08	0.01	0.02
25	7A	589	99,445,664	169,125.30	0.07	0.09	0.08	0.08	0.09	0.02	0.02
26	7B	277	85,477,300	309,700.40	0.08	0.09	0.10	0.08	0.10	0.01	0.02
27	7C	288	78,312,350	272,865.30	0.1	0.11	0.12	0.10	0.13	0.02	0.04
28	7D	479	55,715,643	116,559.90	0.1	0.14	0.12	0.13	0.12	0.01	0.02
		9622	1,729,471,880		0.19	0.22	0.23	0.20	0.23	0.03	0.05

Supplementary Table S3**.** Type-A additive correlations among trials for five traits based on a multi-environmental trial analysis model that uses all information. AWT, average fruit weight (g); EMY, early marketable yield (g per plant); SSC, soluble solids content (°Brix); TC, proportion of total culls (%); TMY, total marketable yield (g per plant).

Trait	Trial	T2	T4	T6	T8	T10
AWT	T2	1	0.962	0.987	0.939	0.950
	T4		1	0.965	0.956	0.998
	T6			1	0.945	0.951
	T8				1	0.951
	T10					1
		T2	T4	T6	T8	T10
EMY	T2	1	0.801	0.986	0.990	0.997
	T4		1	0.692	0.878	0.843
	T6			1	0.953	0.972
	T8				1	0.998
	T10					1
		T2	T4	T6	T8	T10
TC	T2	1	0.819	0.902	0.791	0.716
	T4		1	0.987	0.952	0.987
	T6			1	0.942	0.948
	T8				1	0.937
	T10					1
		T2	T4	T6	T8	T10
SSC	T2	1	0.995	0.972	0.987	0.914
	T4		1	0.99	0.966	0.900
	T6			1	0.921	0.866
	T8				1	0.917
	T10					1
		T2	T4	T6	T8	T10
TMY	T2	1	0.999	0.902	0.887	0.915
	T4		1	0.880	0.863	0.895
	T6			1	0.999	0.999
	T8				1	0.998
	T10					1

Supplementary Table S4**.** Forward predictive ability (PA) for five traits estimated using GBLUP, for pairs of trials making use of all individuals including varieties and advanced selections in common among each pair of trials. AWT, average fruit weight (g); EMY, early marketable yield (g per plant); SSC, soluble solids content (°Brix); TC, proportion of total culls (%); TMY, total marketable yield (g/plant).

TRN	TST	AWT	EMY	SSC	TC	TMY
T2	T4	0.53	0.29	0.40	0.32	0.39
T2	T6	0.39	0.32	0.37	0.25	0.36
T2	T8	0.44	0.21	0.40	0.19	0.24
T2	T10	0.42	0.37	0.35	0.22	0.39
T4	T6	0.38	0.17	0.40	0.36	0.29
T4	T8	0.41	0.24	0.38	0.29	0.24
T4	T10	0.44	0.25	0.40	0.33	0.33
T6	T8	0.42	0.29	0.35	0.21	0.32
T6	T10	0.42	0.39	0.27	0.28	0.46
T8	T10	0.51	0.39	0.41	0.33	0.46
